# Evaluating the accuracy of AIM panels at quantifying genome ancestry

**DOI:** 10.1186/1471-2164-15-543

**Published:** 2014-06-30

**Authors:** Jacobo Pardo-Seco, Federico Martinón-Torres, Antonio Salas

**Affiliations:** Unidade de Xenética, Departamento de Anatomía Patolóxica e Ciencias Forenses, and Instituto de Ciencias Forenses, Grupo de Medicina Xenómica (GMX), Facultade de Medicina, Universidade de Santiago de Compostela, 15872 Santiago de Compostela, Galicia, Spain; Grupo de Investigación en Genética, Vacunas, Infecciones y Pediatría (GENVIP), Hospital Clínico Universitario and Universidade de Santiago de Compostela (USC), Santiago de Compostela, Galicia, Spain; Unidad de Emergencias Pediátrica y Cuidados Intensivos, Departamento de Pediatría, Hospital Clínico Universitario de Santiago, Santiago de Compostela, Galicia, Spain

**Keywords:** Genomics, SNPs, AIMs, Ancestry

## Abstract

**Background:**

There is a growing interest among geneticists in developing panels of Ancestry Informative Markers (AIMs) aimed at measuring the biogeographical ancestry of individual genomes. The efficiency of these panels is commonly tested empirically by contrasting self-reported ancestry with the ancestry estimated from these panels.

**Results:**

Using SNP data from HapMap we carried out a simulation-based study aimed at measuring the effect of SNP coverage on the estimation of genome ancestry. For three of the main continental groups (Africans, East Asians, Europeans) ancestry was first estimated using the whole HapMap SNP database as a proxy for global genome ancestry; these estimates were subsequently compared to those obtained from pre-designed AIM panels. Panels that consider >400 AIMs capture genome ancestry reasonably well, while those containing a few dozen AIMs show a large variability in ancestry estimates. Curiously, 500-1,000 SNPs selected at random from the genome provide an unbiased estimate of genome ancestry and perform as well as any AIM panel of similar size. In simulated scenarios of population admixture, panels containing few AIMs also show important deficiencies to measure genome ancestry.

**Conclusions:**

The results indicate that the ability to estimate genome ancestry is strongly dependent on the number of AIMs used, and not primarily on their individual informativeness. Caution should be taken when making individual (medical, forensic, or anthropological) inferences based on AIMs.

**Electronic supplementary material:**

The online version of this article (doi:10.1186/1471-2164-15-543) contains supplementary material, which is available to authorized users.

## Background

With the publication of the Human Genome Project (http://www.ornl.gov/sci/techresources/Human_Genome/home.shtml) in 2000 and the pioneering high-throughput single nucleotide polymorphism (SNP) genotyping projects (such as HapMap; http://www.hapmap.org) our perception of human genome has changed, as well as our understanding of human evolution and genome ancestry. The term ancestry refers to “*the origin or background of something*” (http://oxforddictionaries.com). Accordingly, in human genetics, ancestry is generally understood as the origin or background of our genomes. However, the question is far from trivial. Considering the way in which the DNA material is inherited through generations, most of it from both parents (the exception being the uniparental markers), entire blocks of our genome can have different ancestral origins. In the words of Svante Pääbo, “*to understand what make us unique, both as individuals and as a species, we need to consider the genome as a mosaic of discrete segments, each with its own unique history and relatedness to different contemporary and ancestral individuals*” [1].

Although genetic variation in humans shows gradients of allele frequencies extending over the entire world (within and among continents or among groups of individuals [2]), there is empirical evidence indicating that the most contrasting genomic patterns of diversity in humans occur at an inter-continental level; e.g. Africa, Europe, and Asia (often erroneously interpreted as genetic support for “races” [2]). The best way to characterize these continental patterns (as discrete clusters of variation) is by examining them to a genomic scale, given that single locus could not necessarily capture the global genomic scenario. With the arrival of new genotyping technologies and large-scale genomic projects, it is now possible to measure genomic ancestry using large genome-wide SNP panels or, more recently, next generation sequencing data (NGS; e.g. http://www.1000genomes.org) [3, 4]. However, this genomic approach is not always cost-effective and it can also represent a handicap in particular scenarios (low amount and/or degraded DNA; e.g. population and forensic routine casework). Alternatively, ancestry can be estimated using a selected number of SNPs ranging rom a few dozens to several hundreds; this option has been favored in different areas of biomedical research, including case-control association studies of complex disease (e.g. admixture mapping) [5–8], human population studies [9–11], and forensic genetics and police investigation [12–14]. The selected SNPs are commonly known as Ancestry Informative Markers (AIMs) and received this name because they exhibit large differences in allele frequencies between populations from different geographical or ethnic groups. By genotyping a number of AIMs, it seems possible to estimate the most likely geographical or ethnic origin of a given genomic profile, or to ascertain what proportion of ancestry in this profile is derived from different geographical regions or source populations.

Measuring ancestry is important in biomedical studies for a number of reasons. For instance, it has been demonstrated that population stratification represents an important confounding effect in case-control association studies of complex and multi-factorial diseases [15–19]. Estimating ancestry using AIMs panels can be used in these studies to control for population sub-structure in medical studies. Some companies have developed commercial kits (http://www.illumina.com/products/dna_test_panel.ilmn) aimed at measuring the ancestry of samples as a screening method before proceeding with their high-throughput genotyping or massive parallel sequencing.

The search for autosomal ancestry has also been a focus of attention in the forensic community [13, 20, 21]. Forensic geneticists have to deal with evidentiary samples containing little amounts of, and/or poorly preserved, DNA. In these cases, the limited amount of DNA available often allows a single PCR reaction only or, in cases where more DNA is available, it is generally preferred to preserve it in order to allow a second and independent test in a different laboratory. Forensic geneticists have also designed their own panels of AIMs allowing estimation of ancestry based on single-plex assays [13, 21].

At the same time, many private companies offer direct-to-consumer-tests (DTCT) specifically designed to measure ancestry [22, 23]. Although most of these tests do not aim to provide specific information about disease conditions, in reality they could reveal information relevant for the customer’s health. This is due to the fact that there are health disorders that can be more highly correlated with certain ancestries than others. However, the accuracy of DTCT has been questioned on several grounds. For instance, these companies often offer only to genotype the uniparental markers (the mitochondrial DNA [mtDNA] and/or the Y-chromosome [23]); however, these markers behave as single locus and therefore can only reflect a very tiny portion of the genomic individual ancestry [24, 25]. Ancestry inferences made using autosomal markers have been conflicting too [26].

Most of the AIM panels available in the literature have been designed by way of selecting SNPs from large genomic databases (e.g. HapMap) showing skewed population frequencies between the ancestral populations targeted. Usually, researchers do not evaluate the amount of genetic informativeness provided individually by the selected SNPs. An exception is the study by Galenter et al. [5], who used a multi-step algorithm that weighs the amount of information provided by their AIMs regarding the ancestral populations being considered.

The ability of an AIM panel to measure ancestry is generally evaluated empirically, that is, by examining its performance on a given set of DNA samples for which a given ancestry is already assumed. Several statistical techniques (such as principal component analysis [PCA], and admixture analysis) are then used to evaluate their efficiency. An AIM panel is generally considered to be efficient if e.g. it can differentiate the targeted populations in the Euclidian space represented by two or three principal components (PC) or if the inferred ancestry is consistent with some expectation (e.g. self-reported ancestry). The majority of the panels are designed with the aim of distinguishing main continental groups (e.g. Africans, Asians, Europeans, Americans) owing to the known difficulties of using small SNP panels to classify individuals when they belong to closely related populations.

The number of SNPs incorporated into AIM panels varies from a few dozens to a few hundreds (Table 1); and this number is generally constrained by the genotyping technique employed. For instance, most of the techniques allow genotyping only a few dozen SNPs in a single PCR reaction (e.g. SNaPshot [13], mass array spectrometry [27]). Although it seems reasonable to consider that the number of AIMs in a panel could be relevant when estimating ancestry, a comprehensive evaluation of this factor has not been carried out to date. Questions arise too about how many autosomal markers would be needed in scenarios of population admixture, where the use of a dense panel of AIMs could be even more important in order to better represent the admixed profiles. Difficulties in correctly estimating genome ancestry could also derive from the application of an AIM panel to a set of samples different from that used during the SNP selection process and the training tests (‘lack of portability’ [28]). At the same time, using mtDNA and Y-chromosome markers to measure genome ancestry could be justified in some genetic contexts [29], but not when trying to infer global individual genome ancestry [24].

**Table 1:** **Corresponding ancestry estimates in three continental HapMap groups, CEU (Europe), CHB (East Asia), and YRI (Africa) using different SNP sets**

			CEU				CHB				YRI			
	SNPs	Training set populations	%	SD	95% CI	Range	%	SD	95% CI	Range	%	SD	95% CI	Range
**Genome Ancestry**	1,440,616	AFR/ASI/EUR	100	0.1	100-100	100-100	100	0	100-100	100-100	100	0	100-100	99.4-100
**10,000 rSNPs** ^**1**^	10,000	AFR/ASI/EUR	99.7	0.7	99.9-100	98.5-100	100	0.2	99.9-100	98.6-100	99.9	0.3	99.5-99.9	96.9-100
**1,000 rSNPs** ^**1**^	1,000	AFR/ASI/EUR	97.0	3.5	98.1-99.4	91.0-100	98.8	2.2	97.7-98.9	91.7-100	98.3	2.2	96.1-98.0	87.0-100
**500 rSNPs** ^**1**^	500	AFR/ASI/EUR	94.8	1.2	96.5-98.5	86.1-100	97.5	3.6	95.8-97.8	86.8-100	96.8	3.6	93.3-96.2	80.4-100
**GAL**	446	AFR/AME/EUR	92.7	3.1	91.9-93.6	86.6-100	96.0	3.0	95.1-96.8	89.5-100	99.1	1.2	98.8-99.4	95.8-100
**ILU**	360 (310)^2^	CEU/CHB + JPT/YRI	87.8	4.7	86.5-89.1	73.9-98.1	97.0	2.5	96.3-97.7	90.1-100	98.7	1.7	98.2-99.2	92.9-100
**HAL**	176^3^ (162)^2^	AFR/AME/EUR/ASI	87.0	7.2	85.0-89.0	70.2-100	93.9	5.1	92.4-95.3	77.6-100	96.2	3.7	95.2-97.2	86.6-100
**KOS**	128	AFR/ASI/EUR/ASI/SAS/AME/MEX/PRI	87.4	6.5	85.6-89.2	73.4-100	90.7	5.4	89.2-92.2	77.9-100	97.9	2.8	97.1-98.7	90.6-100
**NAS**	93	OCE/ASI/AFR/SAM/EUR	87.5	7.4	85.4-89.5	70.2-100	89.0	6.1	87.3-90.6	77.0-100	97.7	3.2	96.8-98.6	88.1-100
**PHI**	34 (27)^2^	AFR/EUR/ASI	93.8	8.2	91.5-96.1	67.5-100	92.7	8.3	90.4-95.0	73.6-100	90.1	9.0	87.6-92.5	65.0-100
**COR**	24 (23)^2^	SAM/EUR/AFR	90.9	9.7	88.2-93.5	58.4-100	89.5	12.7	86.0-93.0	37.5-100	92.7	8.1	90.5-95.0	57.7-100
**LAO**	10	AFR/EUR/ASI/AME	83.6	14.5	79.6-87.7	48.5-100	85.9	19.0	80.7-91.2	30.4-100	81.0	16.5	76.4-85.5	33.9-100

CEU column shows the percentages (%) of European ancestry in CEU, CHB column shows the percentage of Asian ancestry in CHB, and YRI shows the percentage of African ancestry in YRI. For each population group the table shows also the standard deviations (SD), 95% confidence intervals (95%CI), and ranges (minimum-maximum values). Genome ancestry refers to the ancestry measured using the full set of SNPs in HapMap. Training set populations refer to the population groups used to design the AIM panels. AFR: Africa, ASI: Asia, SAS: South Asia, EUR: Europe, AME: America, MEX: Mexico, SAM: South America, OCE: Oceania, PRI: Puerto Rico.

^1^Averaged values over all the re-samples.

^2^Number of SNPs indicated in round brackets are those contained in the HapMap database.

^3^Halder et al. [37] refer to 170 AIMs; however, their supplementary data file refers to 176 AIMs.

## Methods

### Population samples

The HapMap SNP database was retrieved from its repository (http://hapmap.ncbi.nlm.nih.gov). This database contains 1,440,616 SNPs genotyped in a total of 1,218 individual samples belonging to the following main continental groups: 472 Africans, 58 Americans, 101 Central-South Asians, 364 East Asians and 223 Europeans. Unless specified, for most of the simulation experiments, only three populations representing the main continental groups were taken from the full HapMap data, namely CEU (European ancestry), CHB (East Asian ancestry) and YRI (African ancestry), with 50 individuals in each group. This decision was based on the fact that most of the AIM panels available were designed to identify ancestry from main population groups.

For some simulation experiments, we created artificial scenarios of admixture by mixing at random the same proportion of SNPs from the following three HapMap datasets: CEU, CHB, and YRI. Therefore, the expected genome admixture in these artificially created hybrid genomic profiles (henceforth referred to as the “AA-genomes”) is 1/3 of ancestry from each of the main continental groups (Asia, Europe, and Africa).

### Sample size and ancestry estimates

The dependence of ancestry inference on sample size was estimated through simulation experiments using a similar procedure to that in Heinz et al. [30]. In brief, for each of the three main continental populations, we randomly selected 1,000 sub-samples of variable sizes (from five to 40 profiles; in stepwise increments of five and taken without replacement). Thus, for example, we obtained 1,000 sub-samples of size five, 1,000 sub-samples of size ten, and so on until a maximum sample size of 40. For each of the sub-samples we computed ancestry proportions as indicated below. Continental ancestry was estimated as the mean value obtained for the 1,000 sub-samples in each sample window, and bootstrapping intervals were built accordingly.

### Statistical analysis

The software Admixture v. 1.22 [31] was used to estimate individual and population ancestries. This software was run using default parameters. Cross validation errors were obtained from Admixture in order to determine the most likely *K* value (*K* indicating the number of inferred clusters showing the lowest cross validation error).

PLINK v.1.07 [32] was used to obtain Identity-By-State (IBS) values between individuals, and IBS values were used to carry out two-dimensional PCA. PLINK was used with default settings. Only when calculating the effect of population sample size on the estimation of ancestry, individual profiles with missing data >10% were filtered out (call rates <90% could be critical when dealing with AIM panels containing low number of SNPs).

Locus specific branch length (LSBL) statistics was estimated using pairwise F_ST_ distances as carried out in Shriver et al. [33]. LSBL aims to assist in the selection of AIMs in panels taking into account their level of individual informativeness with regards to the classification population sets. F_ST_ values were taken from SPSmart and ENGINES [34, 35].

In-house R 2.13.0 (http://www.r-project.org) and Perl (http://www.perl.org) scripts were used to display results obtained from the different software packages used.

### Pre-designed AIM panels

Ancestry of the selected HapMap datasets was estimated using different AIM panels (Table 1): Corach et al. [36] (COR), Galenter et al. [5] (GAL), Halder et al. [37] (HAL), Kosoy et al. [38] (KOS), Lao et al. [11] (LAO), Nassir et al. [39] (NAS), Phillips et al. [13] (PHI), and the commercial DNA Test Panel from Illumina (ILU; http://www.illumina.com/products/dna_test_panel.ilmn). All of these panels were originally designed to differentiate the three main continental groups (Europe, East Asia and Sub-Saharan Africa), some of them also including other ancestral groups (see Table 1).

## Results

### Estimating genome ancestry

**Figure 1:**
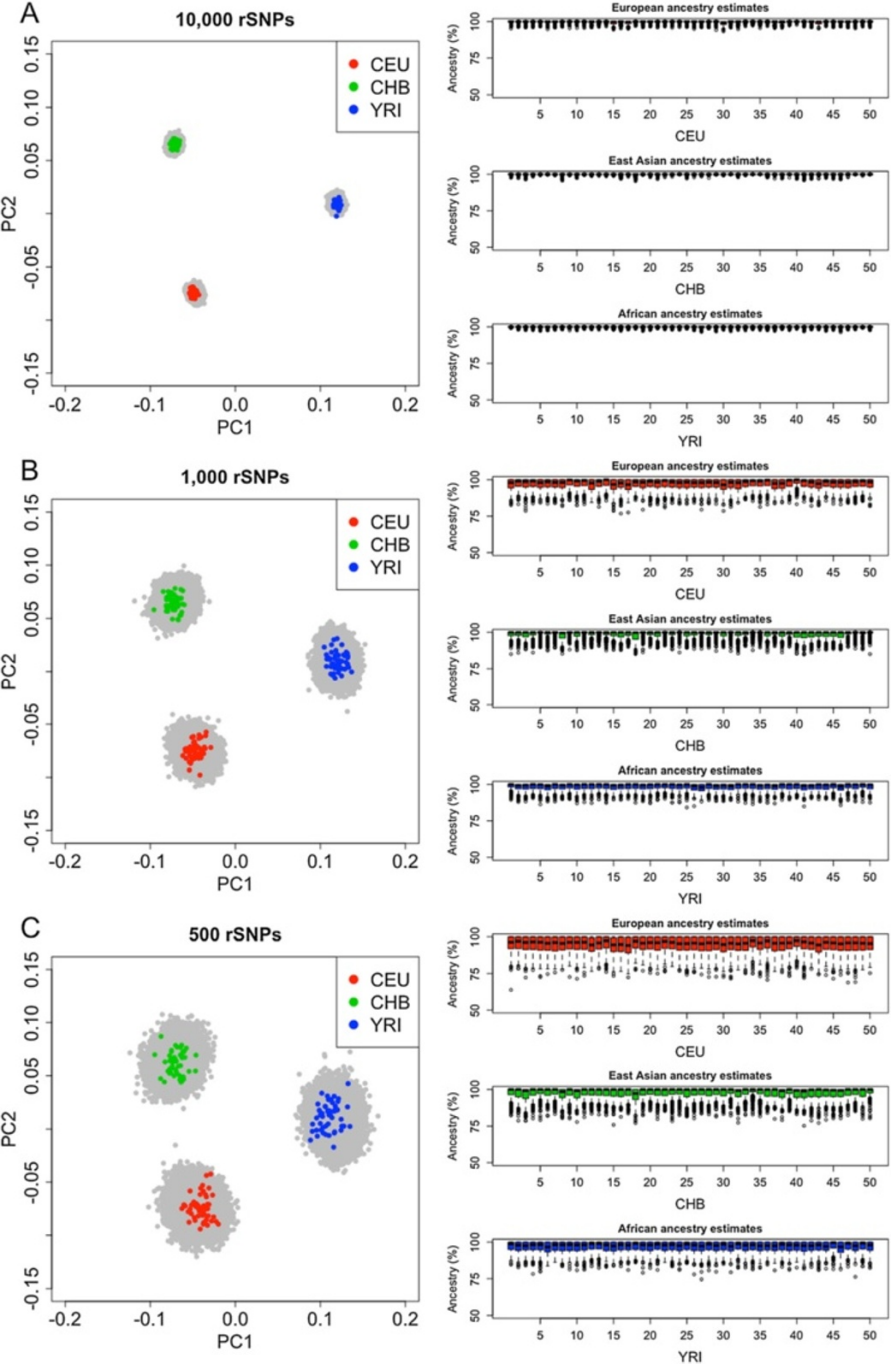
**PCA plots of YRI, CHB and CEU carried out using 500 re-samples of rSNPs from HapMap taking at random 10,000 (1A), 1,000 (1B) and 500 (1C) SNPs.** Only one of the re-samples is highlighted in color; the results for the remaining 499 re-samples are indicated in grey with the aim of illustrating the variability on ancestry estimates associated to the random sub-sets of SNPs. The box-plots (right panels) indicate the European, Asian and African ancestries in CEU, CHB, and YRI, respectively, as obtained in the different re-samples. A statistical summary of these box-plots is given in Table 1.

The genome-wide set of SNPs in HapMap can be used to estimate (global) genome ancestry of continental groups. Admixture analysis shows an optimum value of *K* = 3 when considering CEU, CHB, and YRI. Each individual profile received virtually 100% of the expected genomic ancestry in one cluster (in agreement with their geographic origin/self-declared ancestry); that is, for example, a Yoruban profile receives a ~100% membership in a cluster that groups all samples of African ancestry (Additional file 1).A simulation experiment was carried out by selecting subsets of random SNPs (henceforth rSNPs) from the whole HapMap database and obtaining estimates of ancestry from these subsets. This procedure allows us to investigate the extent to which the estimation of genome ancestry degenerates when using decreasing amounts of SNPs. Panels of 10,000, 1,000, and 500 rSNPs where randomly selected; each panel size was sampled 500 times each in order to account for sampling variability. The simulations indicate that the inferred genome ancestry degenerates slightly as fewer rSNPs enter in a set (Figure 1). However, ancestry estimates using panels of 500 rSNPs approach well the genome ancestry (although the estimates show a moderate dispersion).

The estimates above were obtained considering the three main continental groups: Europe, Asia, and Africa. However, the number of SNPs needed to infer ancestry strongly depends on the evolutionary relatedness of the populations being considered: the closer the population under study, the larger the number of SNPs needed. The PCA plot in Additional file 2 indicates that the whole set of SNPs in HapMap clearly separates East Asian populations CHB + CHD (Chinese) from JPT (Japanese). However, using panels of 100,000, 50,000,10,000, 1,000 and 500 rSNPs, differentiating these two populations groups becomes increasingly difficult; see e.g. the overlapping patterns of profiles in the PCAs of Additional file 2 when using 500 rSNPs. In population scenarios considering very closely related groups, the whole power of a genome-wide dataset would be needed in order to differentiate populations; e.g. see the case for European populations in Novembre et al. [4].

### Pre-designed AIM panels

**Figure 2:**
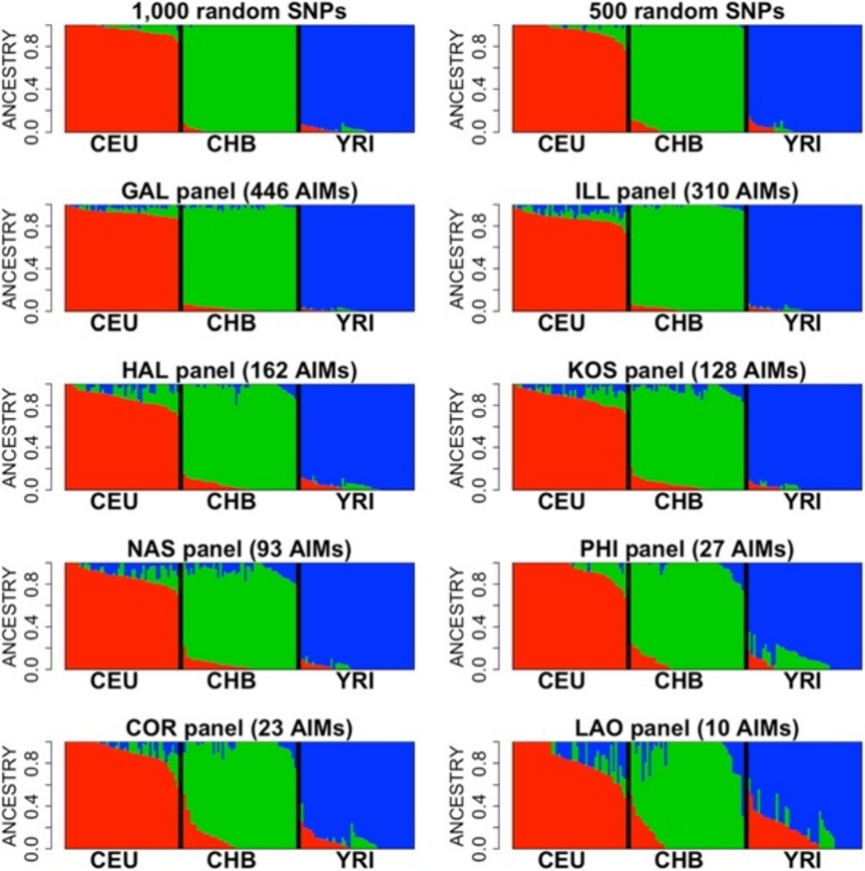
**Bar-plots of ancestry memberships inferred for YRI, CHB, and CEU, considering 1,000 and 500 rSNPs sets (each considering one sample taken at random from HapMap) and the different AIM panels.**

**Figure 3:**
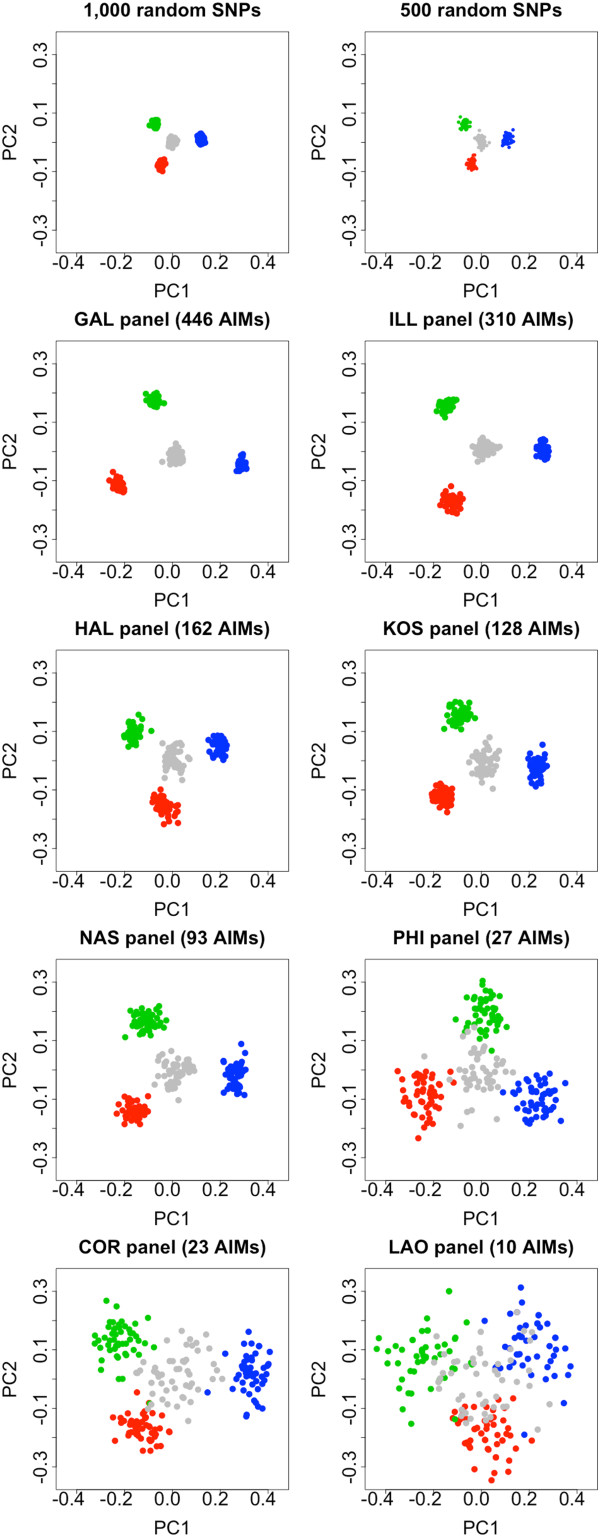
**PCA plots obtained for YRI, CHB, and CEU considering 1,000 and 500 rSNPs (one sample each) taken at random from HapMap as well as different AIM panels.** The inferences carried out on AA-genomes are shown in grey; note that the variation (size of the grey point cloud) increases as fewer rSNPs or AIMs are considered.

Ancestry estimates were obtained using different pre-designed panels and compared to the genome ancestry inferred using sets of 10,000, 1,000 and 500 rSNPs. As shown in the bar-plots in Figure 2, inference of ancestry degenerates as fewer AIMs are considered in the panels. The PCA plots of Figure 3 mirror the same scenario. It is noticeable that the panel of 500 rSNPs yields broadly similar results to the GAL and ILL panels, that is, the panels containing a similar number of AIMs. PCA clearly shows that the panels containing fewer AIMs show more disperse patterns. The LAO panel, which considers only 10 AIMs, represents the most extreme case, showing an extremely large variability (Table 1, Figure 3).

### Measuring informativeness of SNPs in AIM panels

LSBL can be used to measure the informativeness of specific AIMs in the panels and their potential to measure different ancestries when applied to the HapMap populations. To the best of our knowledge, only the GAL panel was designed using LSBL as a criterion to balance the informativeness of the AIMs incorporated in the panels (Table 2). As shown in Additional file 3, all the panels show unbalanced accumulated LSBL values, including GAL. However, both of the panels that contain the largest number of AIMs (GAL and ILL) perform reasonably well when estimating genome ancestry (see above). This evidence suggests that, even though measuring the informativeness of the AIMs would seem a logical way to proceed when designing a panel, ensuring that a large number of AIMs is considered constitutes at least an equally important parameter.

**Table 2:** **LSBL values for the AIMs considered in the different SNP panels and when considering HapMap populations**

	Accumulated LSBL			Average LSBL		
Panel ID	AFR	ASI	EUR	AFR	ASI	EUR
HapMap	143264.06	91184.56	50587.85	0.037	0.024	0.013
GAL	71.08	23.35	46.67	0.159	0.052	0.105
ILU	37.11	29.82	17.28	0.120	0.096	0.056
HAL	13.21	13.90	5.66	0.084	0.089	0.036
KOS	14.18	7.41	9.08	0.111	0.058	0.071
NAS	12.87	6.32	6.58	0.138	0.068	0.071
PHI	3.29	3.08	3.66	0.122	0.077	0.136
COR	4.96	2.41	1.74	0.216	0.105	0.076
LAO	1.31	1.30	0.56	0.131	0.130	0.056
Present study (595 SNPs)	98.38	98.40	98.35	0.165	0.165	0.165

The term “average LSBL” refers to the LSBL accumulated and standardized by the number of AIMs in each panel. LSBL in HapMap was calculated using the HapMap database that considers all African, European and Asian populations together.

We also followed the LSBL criteria for selecting the best AIMs from the HapMap database (as done in Galanter et al. [5]); in each case, the number of simulated AIMs selected was the same as the number used in the different panels tested. Additional file 3 shows that these test panels work better than their pre-designed counterpart panel using analogous continental populations (TSI as representative of Europe; CHD from East Asia and LWK from Africa). However, those containing a higher number of HapMap-AIMs perform much better than those considering lower numbers of SNPs.

### Effect of population sample size on the estimation of ancestry

**Figure 4:**
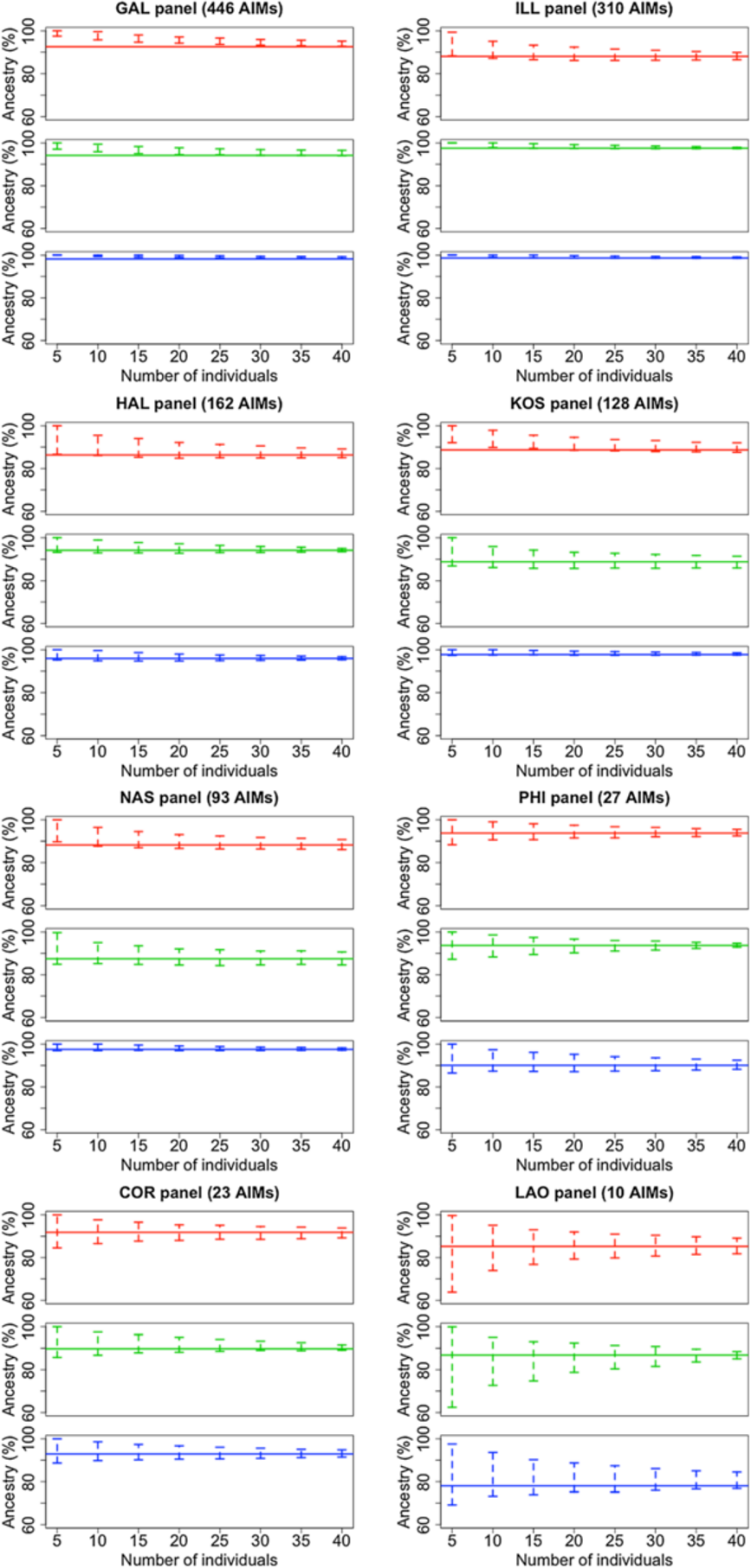
**Effect of sample size on the inference of ancestry using different AIM panels.** The horizontal bar indicates the genome ancestry as estimated using all the HapMap individuals for each (CEU, CHB, YRI) and it marks therefore the value to which all the ancestry estimates from AIM panels should converge. As the number of individuals increases, the estimates of ancestry using the different panels approach the genome ancestry. Color codes are as follows, red: African ancestry; green: Asian ancestry; and blue: European ancestry.

Simulations were carried out in order to estimate the effect of population sample size when inferring genome ancestry using different AIM panels. These analyses are significant because geneticists are often interested in measuring the average genetic ancestry of a given population (see e.g. Heinz et al. [30]), but sample sizes are very low in several studies.As expected, simulations indicate that as the sample size increases the variability of the ancestry estimations decreases (Figure 4). The most remarkable finding is that, for equal sample sizes, the largest AIM panels, GAL and ILL, show lower variability than smaller panels.

### Inferring ancestry in admixed genomes

**Figure 5:**
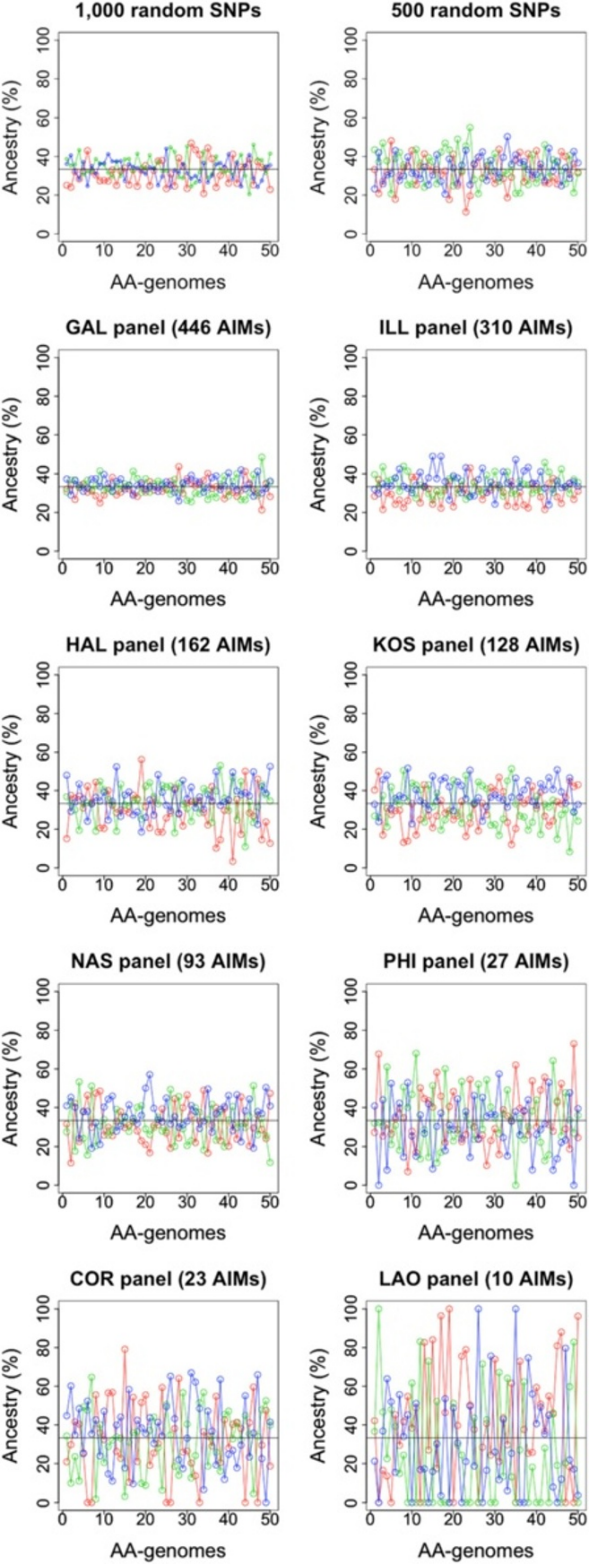
**Estimation of ancestry on AA-genomes using two panels of 1,000 and 500 rSNPs from HapMap and the AIM panels.** The horizontal bar represents the genomic ancestry of AA-genomes that are assumed to have equal ancestry membership in Africa, East Asia, and Europe (~33% each). Color codes for ancestries are as indicated in legend of Figure 4.

The hybrid AA-genomes were created in order to allow some simulations to investigate the number of SNPs needed to capture genome ancestry in admixture scenarios. Figure 5 shows the patterns of ancestry estimated using the different panels and compared to the expected ancestry in these genomes (equal membership in Africa, East Asia and Europe; ~33%). The estimates of individual ancestry are stable and close to expectation when using rSNPs (>1000) and also using GAL (and slightly worse with ILL); while other panels such as PHI, COR and particularly LAO show more arbitrary patterns.

### Quantifying errors in ancestry estimates

**Figure 6:**
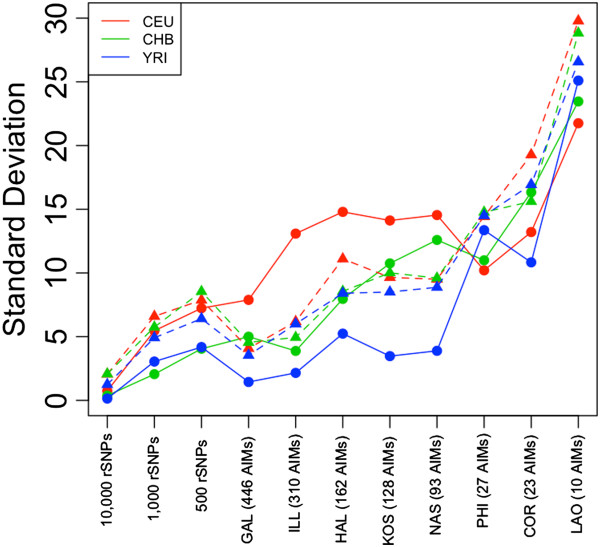
**Error of the different panels in the estimation of genome ancestry for CEU, CHB, and YRI, measured as standard deviations regarding genome ancestry (inferred using the whole HapMap SNP database)** ***versus*** **the different AIM panels.** Solid circles and lines indicate errors on non-admixed genomes, while triangles (and dashed lines) indicate errors on admixed genomes (AA-genomes).

The standard deviation can be used to measure the error of the estimated ancestry obtained from panels, compared to global genome ancestry (Figure 6). The errors in ancestry estimates are more pronounced when the number of rSNPs falls to ~500 SNPs (Figure 6). The ability of panels to capture different proportions of ancestry varies significantly from panel to panel, but the panels with more AIMs (specially GAL and ILL) perform much better than those containing fewer SNPs. For instance, panels PHI and COR show very large variability in ancestry estimates, and this variability is extremely large in the case of LAO (Figure 6). Additional file 4 shows that there is a negative correlation between the number of SNPs in a panel and the error associated to the estimates of ancestry. Furthermore, the error differs regarding the kind of ancestry that is measured: for instance, the panels with the larger numbers of AIMs have more difficulties to measure the European ancestry than the African one. Curiously, the error in ancestry estimates seems to have a more balanced behavior regarding the inferences of the different ancestries in scenarios of admixture (using AA-genomes).

## Discussion

Measuring genome ancestry is an issue of interest in different fields of biomedical research, including case-control association studies, forensic casework and police investigation, and anthropological studies. It is also of interest for private companies, given the growing social interest in knowing more about ancestry coupled with the progressive reduction of the cost of DNA tests.

The present study aims to estimate the number of SNPs needed to reliably infer genome ancestry using unbiased sets of SNPs (rSNPs) and sets of pre-fabricated AIM panels.

The results indicate that 10,000 SNPs selected at random from an individual can be used to infer genome ancestry with negligible error when considering the three HapMap populations CEU, CHB, and YRI. Even so, panels of 500 rSNPs perform reasonably well in this population scenario. Below this number, errors in the inference of ancestry increase noticeably as the number of rSNPs is reduced. As expected, the number of rSNPs needed to infer ancestry strongly depends on the evolutionary proximity of the populations under study. For instance, we made simulations to test the number of rSNPs needed to differentiate ancestry in two different East Asian populations, Chinese and Japanese. Here the number of rSNPs needed to differentiate these populations increases significantly more than one order of magnitude; therefore, the need for searching panels of highly discriminating AIMs is more justified. The distinction between individual ancestries within Asian populations (or other closely related groups) would require genome-wide screenings [4] or very large panels of AIMs (probably containing thousands of SNPs).

During the last few years, several panels of SNPs have been designed in order to estimate ancestry using only a few markers (AIM panels). Analyses were carried out in the present study in order to assess the performance of these panels when applied to three main HapMap continental populations, CEU, CHB, and YRI. The results indicate that inference of ancestry can be seriously compromised when using panels containing small numbers of AIMs. For instance, out of the panels tested in the present study, those showing the best performance are ILU and GAL, that is, those that have more AIMs, while the ones including only a few dozen AIMs show higher errors and variability (Additional file 4).

It is interesting to note that neither GAL nor ILU were specifically conceived to discriminate exactly between the three tested populations from HapMap (Table 1). In fact, the cumulative LSBL value for the three HapMap populations indicates that these AIMs are not balanced for these population groups (Figure 5). Therefore, the good performance of these panels is based to a great extent on the large number of AIMs contained in these panels, and not exclusively on the individual discriminatory power of the selected SNPs.

Of the different AIM panels tested in the present study, only GAL [5] was initially designed using a criterion of SNP informativeness; thus, markers were selected on the basis of balanced cumulative LSBL values in the targeted populations. The present study reveals two limitations in this procedure. First, this method does not specify how many SNPs should enter the ancestry SNP panel; thus, different amounts of SNPs could fit the criteria of similar cumulative LSBL values [5]. Second, it is hard to predict the extent to which the good LSBL characteristics of the AIMs in a panel (in training set populations) can be extrapolated to other population sample sets (which may or may not belong to a closely related geographic/ethnic group). The results of the present study indicate that the best way to ensure the good performance of a panel is to incorporate the largest possible number of AIMs (at least >400 when considering main continental groups).

Our results allow further relevant conclusions. First, inferences related to population demography (e.g. molecular anthropological studies) could be biased if using panels containing a small number of AIMs. Second, DTCT should consider employing panels containing large amounts of markers in order to provide the most accurate service to the public [23]. Third, one of the most important limitations in forensic casework and police DNA investigation is the amount and quality of DNA available from evidentiary samples; here the use of AIM panels could play an important role given that only a limited number of SNPs can enter a single PCR reaction. However, forensic specialists and police investigators should be aware of the limitations of the approach; where possible, a large number of AIMs should be analyzed in order to provide the most precise inferences on the ancestry of evidentiary samples. Inferences of ancestry could be particularly compromised in scenarios of admixture. In such scenarios, SNP coverage can be more crucial given the need to represent the genome more densely than in scenarios of non-admixture (where only one main component has to be measured). The arrival of NGS technologies may help overcome these limitations; see however some caveats in Bandelt and Salas [40].

## Conclusions

Caution should be exercised when inferring ancestry using AIM panels. The concept of ancestry is a complex one and although it can be operational for particular purposes, it can lead to erroneous perceptions of human variability. As stated by Sankar and Cho [41]: “*the appearance of clustering is a function of how populations are sampled, of how criteria for boundaries between clusters are set, and of the level of resolution used. In the same way that the earth can be described by many different kinds of maps* —*from topological to economic*—*so, too*, *can the naturally occurring genetic variation among populations be divided in numerous ways and be made to highlight any chosen similarity or difference”.* This conclusion is particularly important for the general public, who is often not aware of the limitations of ancestry DNA tests; and also in police investigation, where over-interpretation of an ancestry test could have important consequences on the investigation of forensic DNA evidence.

## Electronic supplementary material

Additional file 1: **PCA analysis of three HapMap populations, YRI, CHB, and CEU using all the SNPs in HapMap.** (TIFF 2 MB)

Additional file 2: **PCA plots of East Asian populations using the whole set of SNPs in HapMap and 500 re-samples of rSNPs taking at random 100,00, 50,000, 10,000, 1,000 and 500 SNPs.** As in Figure 2, the profiles that correspond to one of the re-samples are shown in color, while the remaining re-samples are shown in grey. (TIFF 7 MB)

Additional file 3: **The distributions on the left show the cumulative LSBL values for the different AIM panels compared to the distributions generated by the best AIMs in the HapMap datasets (indicated in the figure as “present study”).** The bar-plots on the right mirror the ancestry inferred using these panels on the HapMap populations TSI (representing Europe), CHD (representing East Asia) and LWK (representing sub-Saharan Africa), in order to reflect the ‘portability’ of the different SNP panels in other population groups. (TIFF 8 MB)

Additional file 4: **Correlation values between the standard errors computed when comparing genomic ancestry with the estimated ancestries using different AIM panels, and considering admixed and non-admixed genomes.** These values correspond to the distributions in Figure 6. (DOC 33 KB)
