# Supplementary figures and images for: Evaluating the accuracy of AIM panels at quantifying genome ancestry
Source: BMC Genomics. 2014 Jun 30;15(1):543. doi: 10.1186/1471-2164-15-543 (PMC4101176; doi:10.1186/1471-2164-15-543)

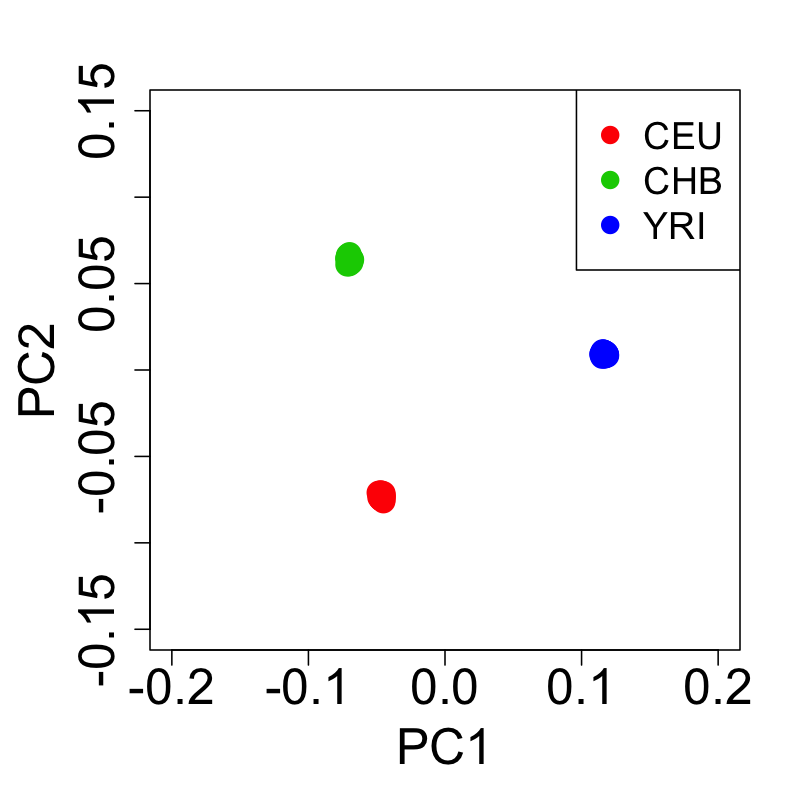

Supplement: Supplementary file 1 — Additional file 1: PCA analysis of three HapMap populations, YRI, CHB, and CEU using all the SNPs in HapMap. (TIFF 2 MB) [file 12864_2014_6238_MOESM1_ESM.tiff]

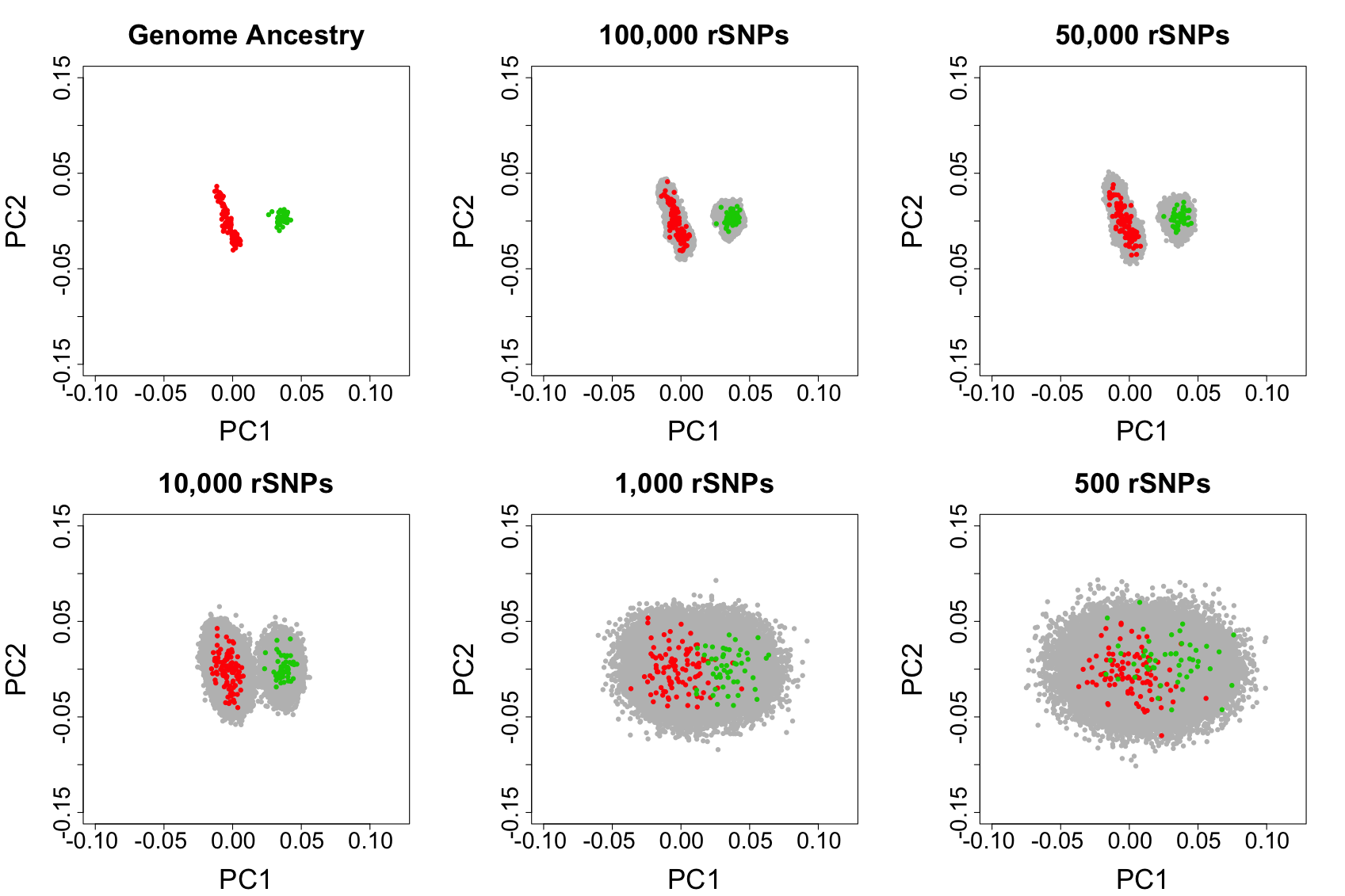

Supplement: Supplementary file 2 — Additional file 2: PCA plots of East Asian populations using the whole set of SNPs in HapMap and 500 re-samples of rSNPs taking at random 100,00, 50,000, 10,000, 1,000 and 500 SNPs. As in Figure 2, the profiles that correspond to one of the re-samples are shown in color, while the remaining re-samples are shown in grey. (TIFF 7 MB) [file 12864_2014_6238_MOESM2_ESM.tiff]

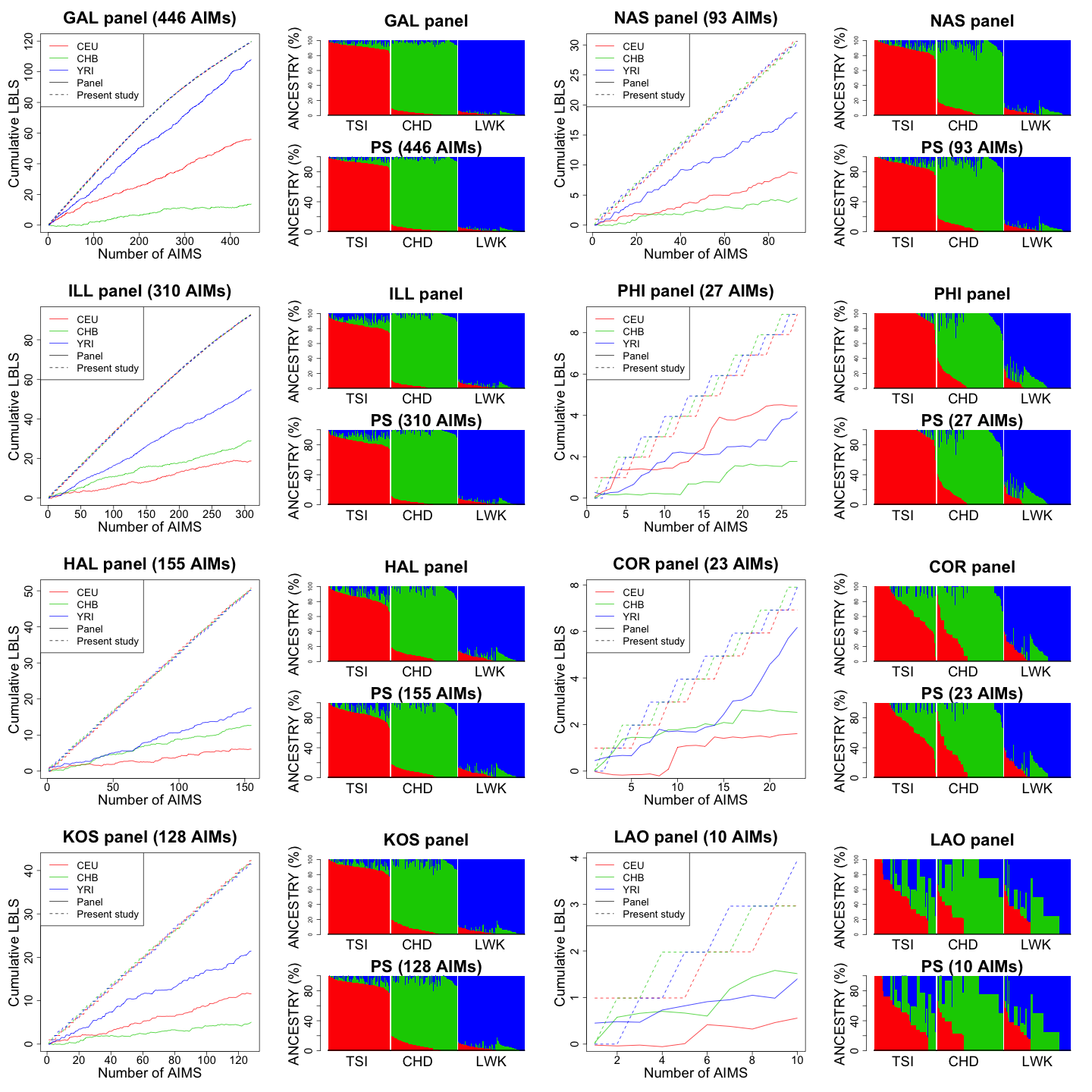

Supplement: Supplementary file 3 — Additional file 3: The distributions on the left show the cumulative LSBL values for the different AIM panels compared to the distributions generated by the best AIMs in the HapMap datasets (indicated in the figure as “present study”). The bar-plots on the right mirror the ancestry inferred using these panels on the HapMap populations TSI (representing Europe), CHD (representing East Asia) and LWK (representing sub-Saharan Africa), in order to reflect the ‘portability’ of the different SNP panels in other population groups. (TIFF 8 MB) [file 12864_2014_6238_MOESM3_ESM.tiff]
