# Supplementary material for: Evaluating the accuracy of AIM panels at quantifying genome ancestry
Source: BMC Genomics. 2014 Jun 30;15(1):543. doi: 10.1186/1471-2164-15-543 (PMC4101176; doi:10.1186/1471-2164-15-543)
Supplement: Supplementary file 4 — Additional file 4: Correlation values between the standard errors computed when comparing genomic ancestry with the estimated ancestries using different AIM panels, and considering admixed and non-admixed genomes. These values correspond to the distributions in Figure 6. (DOC 33 KB) [file 12864_2014_6238_MOESM4_ESM.doc]

**Table S1**. Correlation values between the standard errors computed when comparing genomic ancestry with the estimated ancestries using different AIM panels, and considering admixed and non-admixed genomes (see **Figure 7** for more information). In brackets are the 95%CI’s. *P*-values indicate that, in most instances, correlation is statistically significant (that is, below a significant value of 0.05).

|  | **Correlation** | ***P*-value** |
| --- | --- | --- |
| **CEU** |  |  |
| Non Admixed Genomes | -.572 (-.910, .222) | 0.138 |
| AA-Genomes | -.761 (-.954, -.121) | 0.028 |
| **YRI** |  |  |
| Non Admixed Genomes | -.794 (-.961, -.203) | 0.019 |
| AA-Genomes | -.747 (-.951, -.089) | 0.033 |
| **CHB** |  |  |
| Non Admixed Genomes | -.688 (-.938, .032) | 0.059 |
| AA-Genomes | -.775 (-.957, -.155) | 0.024 |
